# Lower ionospheric resonance caused by Pekeris wave induced by 2022 Tonga volcanic eruption

**DOI:** 10.1038/s41598-024-65929-x

**Published:** 2024-07-16

**Authors:** Hiroyo Ohya, Fuminori Tsuchiya, Tamio Takamura, Hiroyuki Shinagawa, Yukihiro Takahashi, Alfred B. Chen

**Affiliations:** 1 Graduate School of Engineering, Chiba University, Chiba, 263-8522 Japan; 2 Graduate School of Science, Planetary Plasma and Atmospheric Research Center, Tohoku University, Sendai, 980-8578 Japan; 3 Center for Environmental Remote Sensing, Chiba University, Chiba, 263-8522 Japan; 4 International Research Center for Space and Planetary Environmental Science, Kyushu University, Fukuoka, 819-0395 Japan; 5 Graduate School of Science, Hokkaido University, Sapporo, 060-0808 Japan; 6 Department of Physics, National Cheng Kung University, Tainan, 701 Taiwan

**Keywords:** Environmental sciences, Natural hazards

## Abstract

The submarine volcano Hunga Tonga–Hunga Ha’apai erupted explosively on January 15, 2022, offering a unique opportunity to investigate interactions between the atmosphere and ionosphere caused by Lamb and Pekeris waves. However, the resonance of Pekeris waves has not been previously detected. In this study, we applied a multi-point monitoring approach focusing on the lower ionosphere and atmospheric electric field. Here we show observed oscillations of 100–200 s in manmade transmitter signals and the magnetic and atmospheric electric fields, which were caused by Pekeris waves. However, no corresponding changes with the period of 100–200 s in atmospheric pressure due to Pekeris waves were observed on the ground. A simulation of neutral wind revealed Pekeris waves oscillating near the mesopause, suggesting resonance. Therefore, the oscillation in atmospheric electric field is interpreted that the resonance in the lower ionosphere was projected onto the Earth's surface via a global electric circuit.

## Introduction

Hunga Tonga–Hunga Ha’apai (HTHH), a submarine volcano in the southern Pacific Ocean (20.54 °S, 175.38 °W), erupted explosively during 04:00–04:15 UT on January 15, 2022^1,2^, with an estimated volcanic explosivity index of 5–6 (on a scale from 0 to 8), determined according to the ejecta volume, eruptive ash column height, and impulse seismic waves^3^. During this Plinian eruption, the volcanic plume reached a height of ~ 58 km^4^ and the thermal energy released was estimated to range from ~ 1.0 × 10^19^ to 2.8 × 10^19^ J, comparable to the 1991 Pinatubo (~ 1 × 10^19^ J) and 1883 Krakatoa eruptions (~ 3 × 10^19^ J)^5^. Due to the large magnitude of the HTHH eruption, Lamb and Pekeris waves were generated^6^. Lamb waves are acoustic waves that propagate non-dispersively in the horizontal direction near the Earth’s surface, with a phase velocity of ~ 310 m/s^7^. Pekeris waves are resonance oscillations inherent to the Earth’s atmosphere, with a slower phase velocity of ~ 230–245 m/s^6,8^. Although Pekeris waves have been described as the L_1_ mode of Lamb waves^9^, they have an anti-phase between the mesosphere and the stratosphere, whereas Lamb waves are vertically in-phase. The energy of Pekeris waves is enclosed between the stratopause and mesopause, within two atmospheric temperature minima, and the amplitudes of Pekeris waves are higher at the upper stratopause (> 45 km). Pekeris waves had never been observed within the 85 years since their theoretical prediction^10^ until they were recorded by the Himawari-8 geostationary satellite following the HTHH eruption^6^.

**Figure 1:**
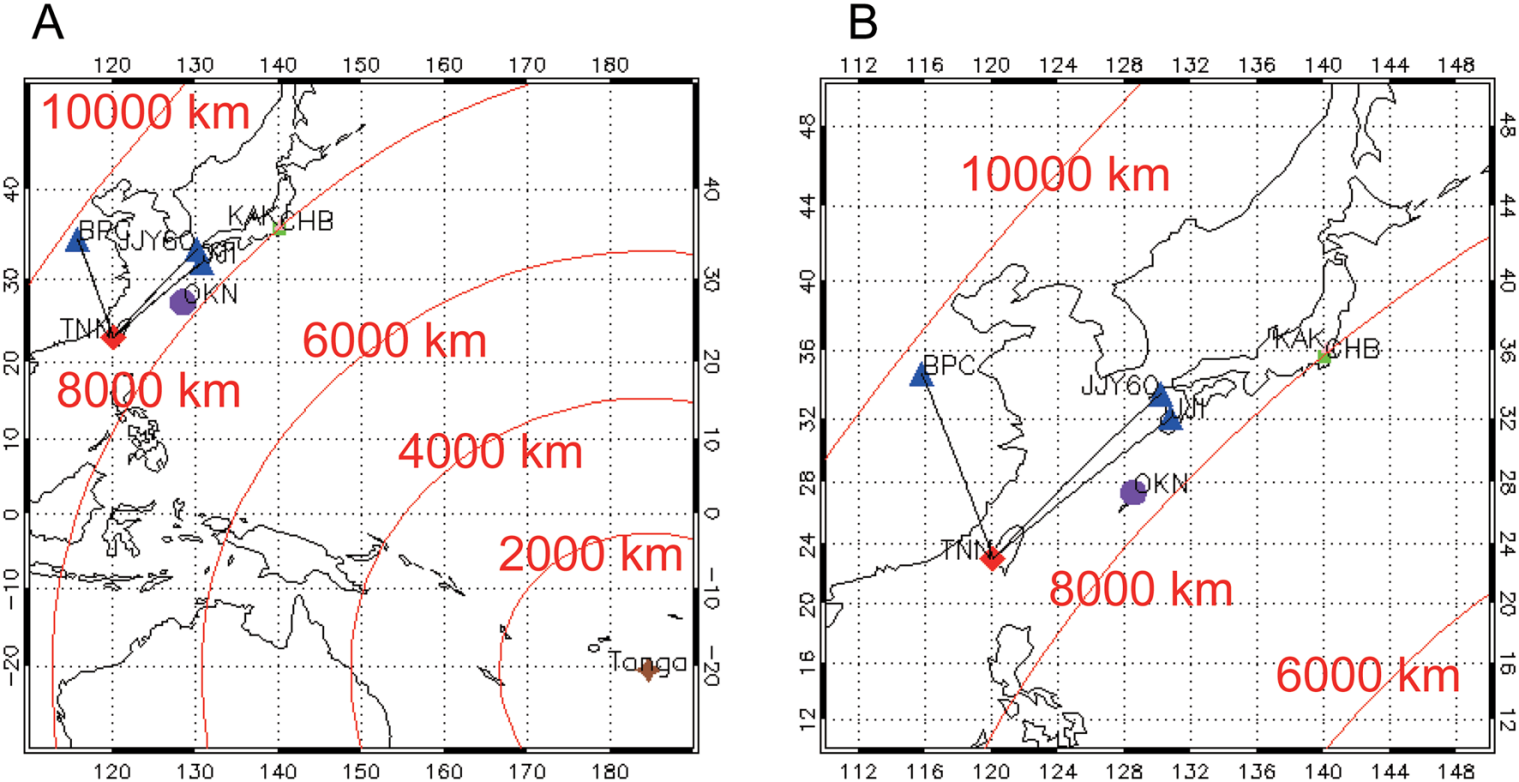
Map of the region influenced by the 2022 Hunga Tonga-Hunga Ha’apai (HTHH) eruption. (**A**) Very low-frequency/low-frequency (VLF/LF) transmitter signals, atmospheric electric field, ground-based magnetic field, and atmospheric pressure data. Brown star indicates the HTHH volcano; blue triangles indicate VLF/LF transmitters; red diamond indicates a receiver; green rectangle indicates location of atmospheric electric field and atmospheric pressure observations at CHB; purple circle indicates location of SORATENA atmospheric pressure observations at OKN; pink triangle indicates the ground-based magnetometer at KAK; red curves indicate distances from the HTHH volcano. (**B**) Enlarged map of an area near observation sites shown in (**A**).

The global electric circuit between the Earth’s surface and lower ionosphere^11^ is driven by an electric power source consisting of lightning discharges, thunderclouds, precipitation, and electrified clouds. In fair weather, the air current flows downward from the ionosphere to the Earth’s surface, whereas it flows upward during thunderstorms. In studies of the global electric circuit, the atmospheric electric field, or potential gradient (*E*_z_), is typically observed using ground-based field mills. After the HTHH eruption, the variation in *E*_z_ observed in the Czech Republic was similar to that in the amplitude of very low-frequency (VLF) transmitter signals, which are used to measure electron density changes in the D-region ionosphere (height, 60–90 km)^12^. Many studies have examined the F-region ionosphere in association with the HTHH eruption, but coupling between the D-region ionosphere and atmosphere due to Lamb and Pekeris waves generated by the HTHH eruption has not been demonstrated. In this study, we investigated variation in the D-region ionosphere using VLF/LF (3–30/30–300 kHz) transmitter signals, as well as *Ez*, ground-based magnetic field data, and ground-level atmospheric pressure data associated with the HTHH eruption (Fig. 1). In particular, the VLF/LF receiver at Tainan, Taiwan (TNN) belongs to the Asia VLF Observation (AVON) system in Southeast Asia^13^.

## Results and discussion

**Figure 2:**
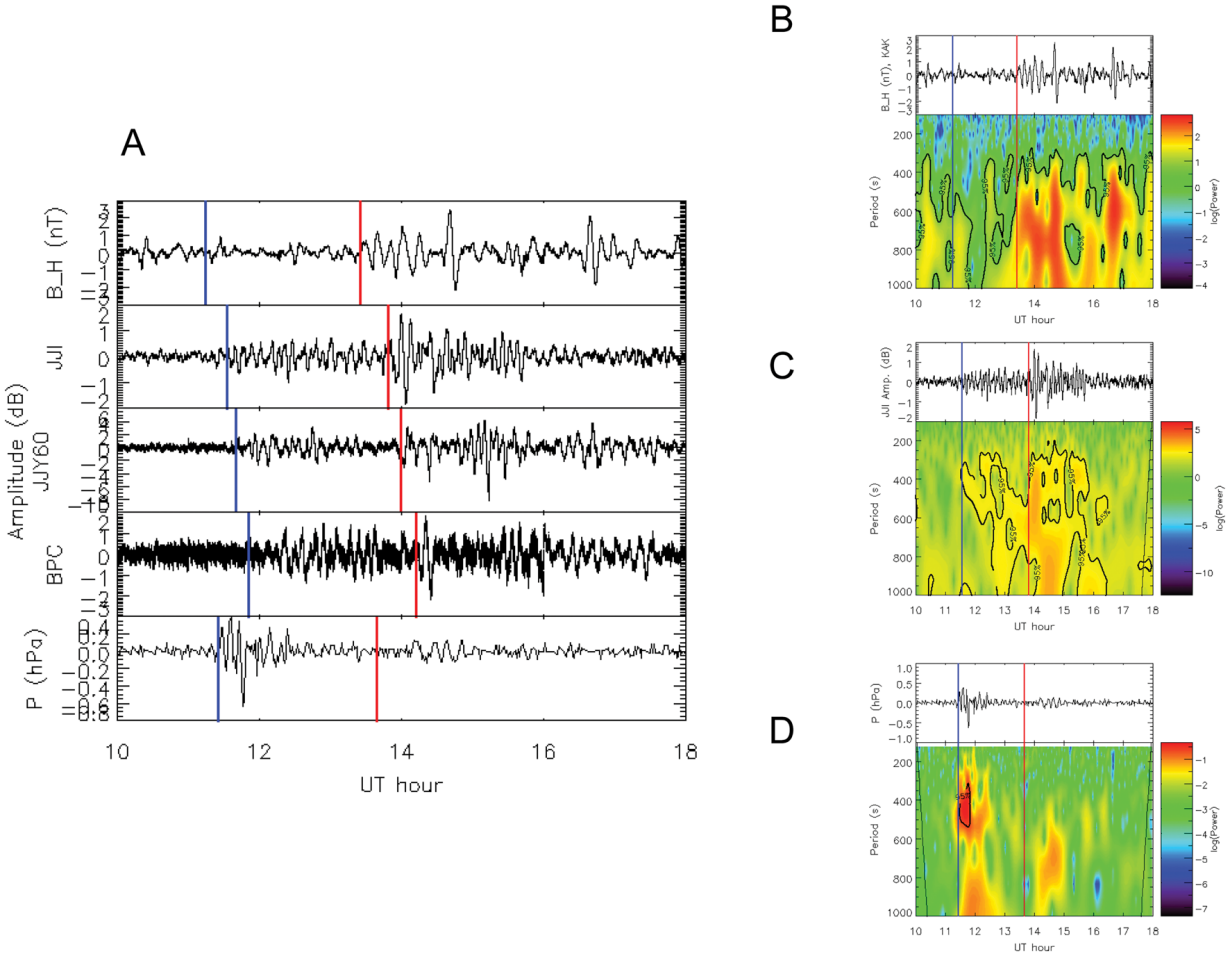
Signatures of Lamb and Pekeris waves in VLF/LF observations (high-pass filter [HPF]: > 1.67 mHz or < 10 min). (**A**) Horizontal (H) component of the magnetic field (*B*_H_) at KAK (top panel), VLF/LF amplitudes for the paths JJI–TNN (second panel), JJY60kHz–TNN (third panel), and BPC–TNN (fourth panel), and atmospheric pressure data at OKN (bottom panel). (**B**) Waveform of *B*_H_ (top panel) and wavelet spectrum (bottom panel). (**C**) Waveform of JJI–TNN amplitude (top panel) and wavelet spectrum (bottom panel). (**D**) Waveform of atmospheric pressure at OKN (top panel) and wavelet spectrum (bottom panel). Blue and red lines indicate Lamb and Pekeris wave arrival times at each site, respectively.

The amplitudes of three VLF/LF paths were processed using a high-pass filter (HPF), with a threshold > 1.67 mHz or < 10 min, and found to vary with the arrival times of both Lamb and Pekeris waves (Fig. 2). Based on the observed VLF/LF amplitudes, the estimated propagation velocities of the Lamb and Pekeris waves were ~ 307 and ~ 235 m/s, respectively, within the ranges of ~ 300–315 m/s and ~ 230–240 m/s reported in previous studies^6,11–13^. The amplitude of the VLF/LF variation due to Pekeris waves (2–4 dB) was higher than that due to Lamb waves (1–2 dB). Similarly, the amplitude of the magnetic field horizontal component (*B*_H_) (HPF: > 1.67 mHz) at Kakioka, Japan (KAK) was lower (± 0.8 nT) when Lamb waves arrived and higher (± 1.5 nT) when Pekeris waves arrived. However, atmospheric pressure (HPF: > 1.67 mHz, *P*) observed by the SORATENA weather sensor at Okinawa (OKN) varied greatly around the Lamb wave arrival time (± 5 hPa), whereas amplitude variation was not observed at the Pekeris wave arrival time. The method for determining the arrival times of the Lamb (the blue vertical line) and Pekeris waves (the red vertical line) is as follows. First, we calculated the horizontal distance from the HTHH volcano to each observation site. For VLF/LF data, we adopted the shortest distance from the HTHH volcano to each propagation path. The distances for the *B*_H_ at KAK, JJI-TNN, JJY60-TNN, BPC-TNN, and *P* at OKN were 7831.9 km, 8167.7 km, 8311.6 km, 8499.9 km, and 8032.3 km, respectively. Next, since the propagation velocity of Lamb waves estimated from VLF data was ~ 307 m/s, we calculated the propagation time of Lamb wave from distance and velocity. Therefore, the Lamb wave should arrive in the order of *B*_H_, *P*, JJI-TNN, JJY60-TNN, and BPC-TNN. The *B*_H_ corresponds to the current in the lower ionosphere, the VLF/LF waves correspond to electron density fluctuations at a reflection altitude of approximately 90 km, and *P* is the atmospheric pressure at the surface of the Earth, so they are arranged in this order from top to bottom in Fig. 2. The period of the VLF variation caused by Lamb waves was the same as that of atmospheric pressure (300–500 s), although the period of *B*_H_ due to Lamb waves (500–1000 s) differed from that of VLF and atmospheric pressure. In contrast, the period of the VLF variation due to Pekeris waves (200–1000 s) for the three paths was similar to that of *B*_H_ (300–1000 s). There was no clear signature in the *P* at the arrival time of the Pekeris wave (red vertical line). This is because the amplitude of Pekeris wave at the Earth’s surface is very small as suggested by theory^10^. At the D-region altitude (60–90 km), which is the reflection altitude of VLF/LF waves, the energy of Pekeris wave is trapped in the atmospheric temperature minimum region (50–110 km height), resonance occurs, and the amplitude of Pekeris wave increases. Therefore, no signatures of Pekeris waves can be seen in *P* at the Earth’s surface, although they can be seen in VLF/LF waves at the D-region altitude. These results demonstrate that Pekeris waves exhibited slight atmospheric pressure fluctuations at the Earth’s surface, although they were amplified at D-region altitudes.

**Figure 3:**
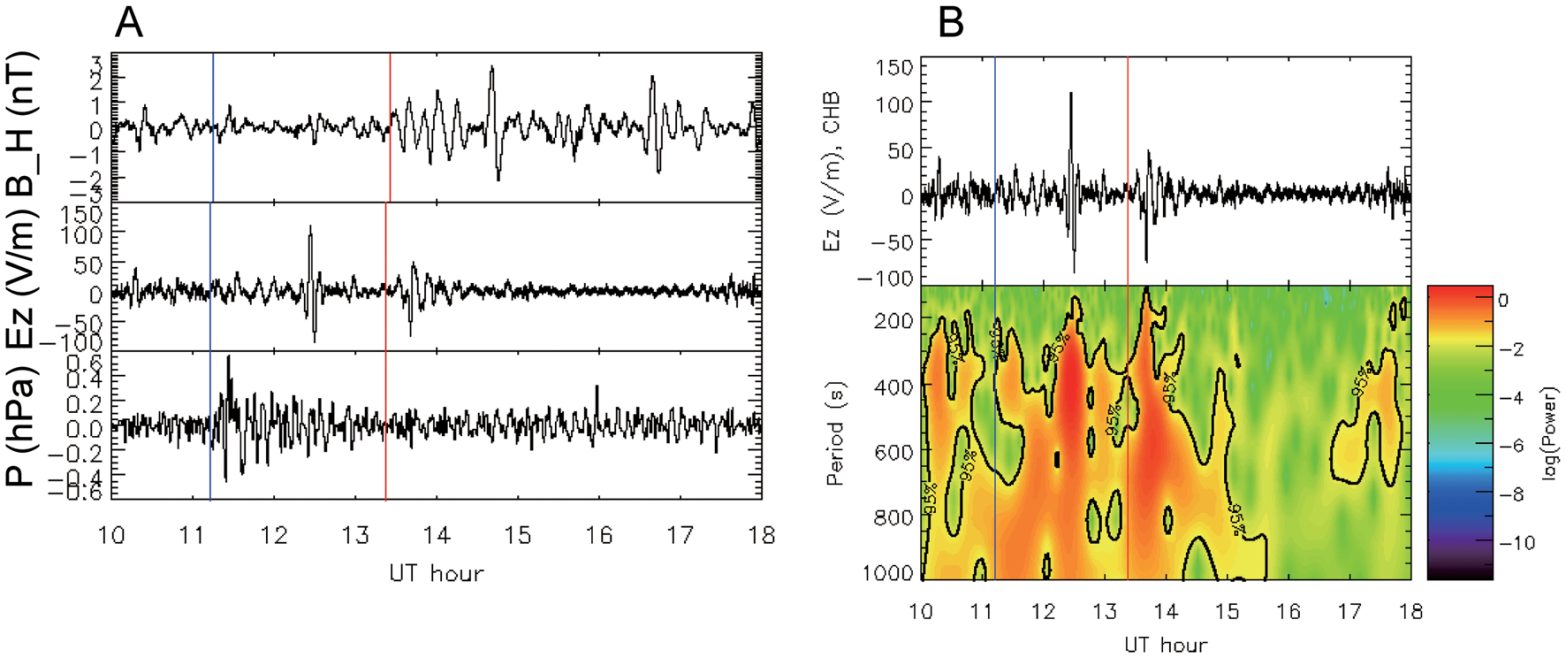
Signature of Pekeris waves in *E*_z_ (HPF: > 1.67 mHz or < 10 min). (**A**) *B*_H_ at KAK (top panel), *E*_z_ at CHB (middle panel), and atmospheric pressure data at CHB (bottom panel). (**B**) Waveform of *E*_z_ at CHB (top panel) and wavelet spectrum (bottom panel). Blue and red lines indicate Lamb and Pekeris wave arrival times at each site, respectively.

Both *E*_z_ (HPF: > 1.67 mHz or < 10 min) at Chiba, Japan (CHB), and *B*_H_ at KAK also varied at the Pekeris wave arrival time, although the amplitudes of these fluctuations were low at the Lamb wave arrival time (Fig. 3). The amplitude of *E*_z_ variation due to Pekeris waves (± 50 V/m) was higher than that due to Lamb waves (± 30 V/m). The periods of the variation in *E*_z_ at CHB due to Lamb and Pekeris waves were 200–1000 and 100–1000 s, respectively, similar to the *B*_H_ range (300–1000 s). In contrast, little variation in atmospheric pressure was observed at CHB due to Pekeris waves, despite large variation at the Lamb wave arrival time (± 0.6 hPa). The amplitudes of Pekeris waves are low on the ground but increase at altitudes above 30 km and become even larger at D-region altitudes^6^. The electron density in the lower ionosphere is influenced by Pekeris waves, leading to changes in the electric current that in turn alter *B*_H_. The air current within the global electric circuit is conveyed by atmospheric ions; therefore, variation in the current in the lower ionosphere is hypothesized to result in an increase or decrease in the number density of atmospheric ions, thereby causing shifts in the atmospheric electric field on the ground.

**Figure 4:**
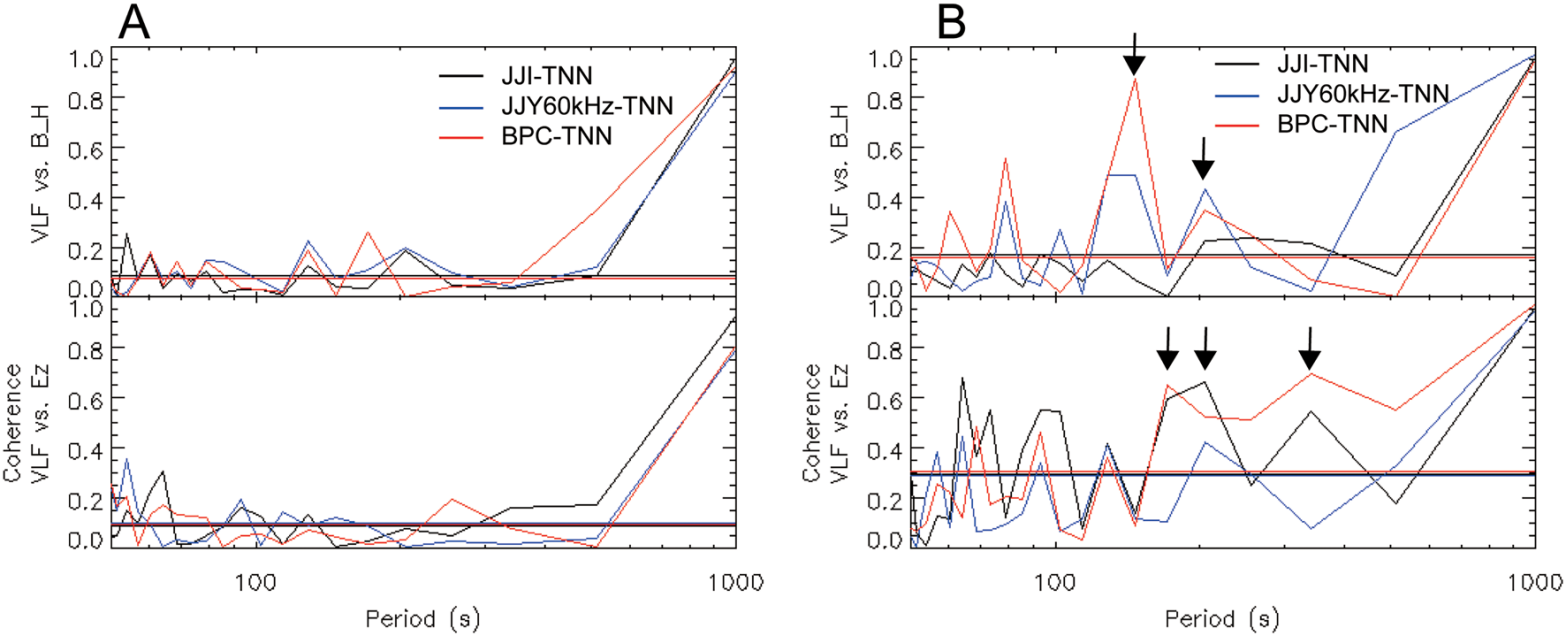
Coherences between VLF/LF amplitudes and other data (ground-based magnetic field at KAK and *E*_z_ at CHB). (**A**) Coherence between VLF/LF amplitudes and *B*_H_ at KAK (top panel) and coherence between the VLF/LF amplitudes and *E*_z_ at CHB (bottom panel) for the Lamb wave. (**B**) Coherence between the VLF/LF amplitudes and *B*_H_ at KAK (top panel) and coherence between the VLF/LF amplitudes and *E*_z_ at CHB (bottom panel) for the Pekeris wave.

**Figure 5:**
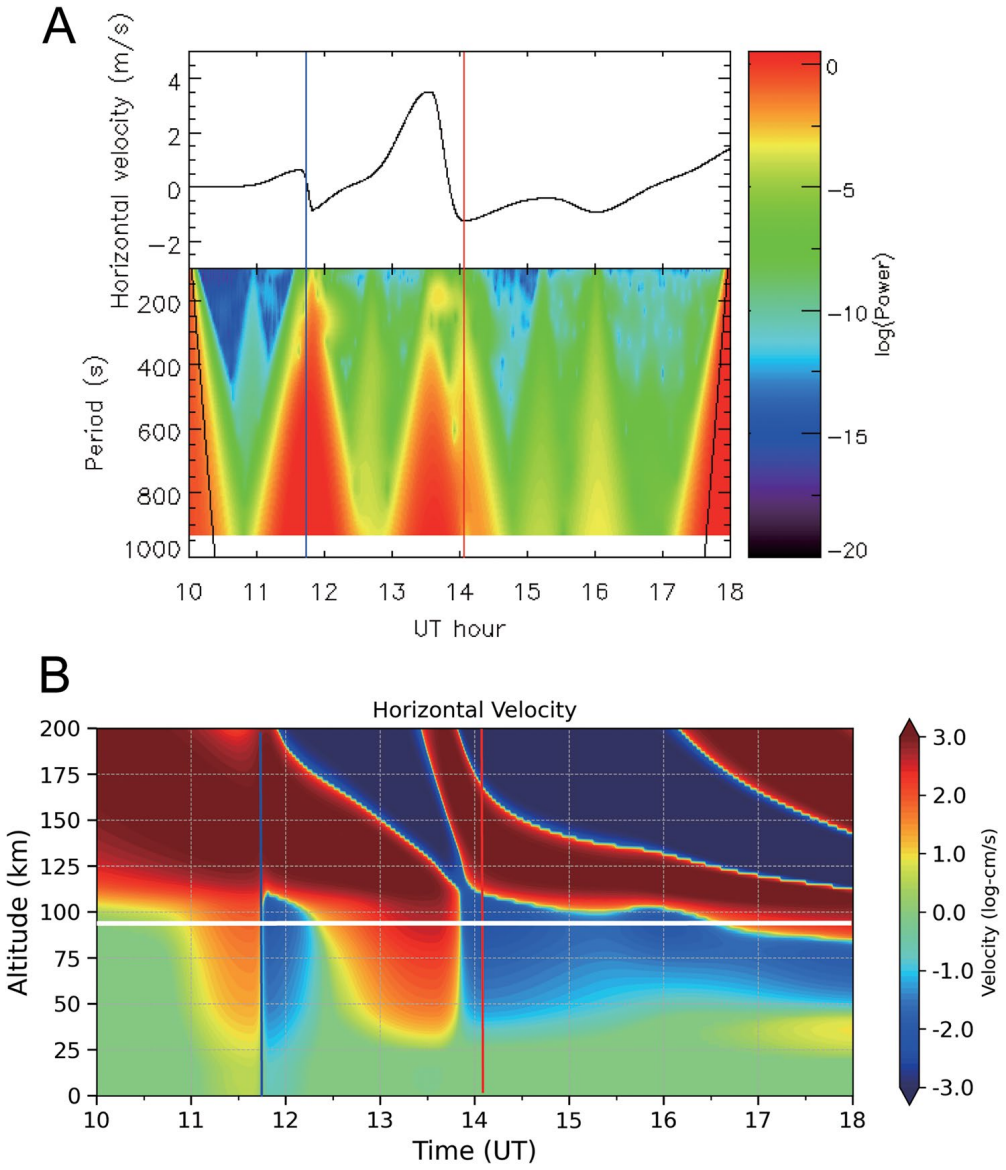
Simulation of horizontal velocity of neutral wind at the midpoint of the JJY60kHz–TNN path (28.3 °N, 125.2 °E) and the wavelet spectrum on January 15, 2022. (**A**) Horizontal velocity at a height of 90 km (top panel) and wavelet spectrum (bottom panel). (**B**) Horizontal velocity below a height of 200 km. Blue and red lines indicate Lamb and Pekeris wave arrival times estimated from observed VLF/LF variations, respectively. White horizontal line indicates an altitude of 90 km. To illustrate small velocities clearly, logarithms of the velocities in the unit of cm/s are taken.

For Lamb waves, coherence values between VLF/LF amplitudes and other data (*B*_H_ and *E*_z_) were < 0.4, although those for Pekeris waves were higher than those for Lamb waves (Fig. 4). The common periods among multiple VLF/LF paths were 146 s (frequency: 6.8 mHz) and 205 s (4.9 mHz) between the VLF/LF amplitudes and *B*_H_, and 171 s (5.8 mHz), 205 s, and 341 s (2.9 mHz) between the VLF/LF amplitudes and *B*_H_ and *E*_z_. In the F-region ionosphere, atmospheric gravity waves (GWs) originating from the HTHH volcano and migrating by Lamb wave had an impact over a long period of time (~ several hours) and over a wide area, causing TID, etc.^15–18^, although duration of GWs migrated by Pekeris wave was short^14^. The Pekeris wave did not has strong effect for the F-region ionosphere. However, in the D-region ionosphere, which is the reflection height of VLF/LF waves, the Lamb and Pekeris waves, rather than GWs, directly changed the electron density. Pekeris wave had a relatively larger amplitude than Lamb wave at this altitude due to acoustic resonance in the neutral wind simulation (Fig. 5). Therefore, for Lamb wave, the *B*_H_ and VLF/LF waves in the lower ionosphere were small and the period of fluctuation was slightly different (Fig. 2), while in the case of Pekeris wave, the *B*_H_ and VLF/LF waves clearly fluctuated with the similar period. The *E*_Z_ at the Earth’s surface was surprisingly similar to the *B*_H_ variation for both Lamb and Pekeris waves. Although atmospheric ions near the Earth’s surface might change due to Lamb wave, it does not seem to result in large changes in the *E*_z_. It is possible that the lower ionosphere was greatly fluctuated by Pekeris wave with increased amplitude due to acoustic resonance, and the fluctuations in the lower ionosphere were projected to *E*_z_ on the Earth’s surface via a global electric circuit. Therefore, these results demonstrate that Pekeris waves cause more D-region ionospheric variation than Lamb waves, and that the D-region ionosphere and *E*_z_ on the ground varied with similar periods via the global electric circuit due to Pekeris waves.

In a neutral wind simulation^14^, Lamb and Pekeris waves were observed at 11:38 UT and 13:33 UT, respectively (Fig. 5). Figure 5A shows that at an altitude of 90 km, the horizontal wind velocity of Pekeris waves is larger than that of Lamb waves. In Fig. 5B, the red areas visible at 10:00–11:40 UT, 11:40–13:30 UT, 13:30–16:00 UT, and 16:00–18:00 UT above 100 km altitudes are GWs caused by the HTHH eruption. This shows that the GWs propagated within the thermosphere and reached the midpoint of the JJY60-TNN path. It looks like this because the first GW has a fast propagation speed of about 750 m/s in the thermosphere, and the subsequent GWs have a slower propagation speed. Below 100 km altitudes, Lamb wave arrived at 11:38 UT and Pekeris waves arrived at 13:33 UT. This can be seen by the sudden change from red to blue at an altitude of 100 km. The simulated arrival time of Lamb waves was in good agreement with the time estimated from VLF/LF observations, although the simulated arrival time of Pekeris waves was earlier than that of VLF waves. These results may have been influenced by background wind, as well as the fact that atmospheric temperature and density inhomogeneities along the VLF/LF propagation paths were not included in the simulation calculations. Lamb and Pekeris wave oscillations with periods of 100–200 s were observed in the horizontal velocity at a height of 90 km. These oscillations were caused by resonance between the stratopause (~ 50 km height) and lower thermosphere. The periods of variation in both the VLF/LF amplitudes and *E*_z_ for Pekeris waves were similar to that of the resonance of 100–200 s shown in the simulation. In the simulation, the horizontal velocity of Pekeris waves (− 1.26 to 3.53 m/s) was higher than that of Lamb waves (− 0.87 to 0.86 m/s), which was in good agreement with the observed amplitudes of variation in VLF/LF waves and *E*_z_, where positive (negative) horizontal velocity values indicate outward (inward) direction relative to the HTHH volcano.

This study is the first to discuss the observation of Pekeris wave resonance in the lower ionosphere in VLF/LF waves, magnetic fields, and the atmospheric electric field. Simulations of neutral wind showed similar oscillations due to Pekeris waves, which were caused by resonance between the stratopause (~ 50 km height) and the lower thermosphere. The atmospheric electric field showed similar oscillations even in ground-based observations, because oscillations near the mesopause were projected onto the Earth’s surface via the global electric circuit.

## Methods

### VLF/LF transmitter signals

The VLF/LF transmitters used in this study were JJI (32.05 °N, 130.82 °E, the frequency: 22.2 kHz), JJY60kHz (33.47 °N, 130.18 °E, 60.0 kHz), Japan, and BPC (34.63 °N, 115.83 °E, 68.5 kHz), China. The receiver was one of AVON and located at Tainan (TNN), Taiwan (23.06 °N, 120.15 °E). The distance between the HTHH and the VLF/LF propagation paths was ~ 8000**–**10,000 km. The receiver detects amplitude and phase of manmade narrowband transmitter signals. These VLF/LF signals received by a monopole antenna are then amplified, filtered, and digitized by the receiving system. The VLF/LF data are 0.1-s sampling. The digital data is automatically transmitted to a processing facility of Tohoku University, Japan. Time synchronization is achieved by a locked-GPS oscillator. To remove noise, we took a 30-s moving average for the raw data.

### Atmospheric electric field data

The atmospheric electric field has been observed with an EFM-100 field mill (Boltek) on the roof of a building on the campus of Chiba University (CHB, 35.63 °N, 140.10 °E), Japan, since 1 June, 2016. The dynamic voltage range is ± 20 kV/m with a 0.5-s sampling time. The cut-off frequency of the low-pass filter is 11 Hz. The distance from the HTHH was ~ 7790 km.

### Atmospheric pressure data

We used two kinds of atmospheric pressure data. The atmospheric pressure data have been observed with a WS601-UMB Smart Weather Sensor (Lufft) at the same place of CHB with atmospheric electric field data. The data were 5-s sampling. The accuracy of the atmospheric pressure was ± 0.5 hPa.

Atmospheric pressure data at Okinawa (OKN, 27.34 °N, 128.57 °E), Japan collected by the SORATENA array were provided by Weathernews Inc. The data was 1-min. sampling. The distance from the HTHH was ~ 8032 km.

### Coherence calculation

Coherence, *coh*(*ω*), is defined by the following formula:

1 $$ {\text{coh}}\left( {\upomega } \right) = \frac{{\left| {{\text{S}}_{{{\text{xy}}}} \left( {\upomega } \right)} \right|^{2} }}{{{\text{S}}_{{{\text{xx}}}} \left( {\upomega } \right){\text{S}}_{{{\text{yy}}}} \left( {\upomega } \right)}} = \frac{{{\text{K}}_{{{\text{xy}}}}^{2} \left( {\upomega } \right) + {\text{Q}}_{{{\text{xy}}}}^{2} \left( {\upomega } \right)}}{{{\text{S}}_{{{\text{xx}}}} \left( {\upomega } \right){\text{S}}_{{{\text{yy}}}} \left( {\upomega } \right)}} $$

where *ω* is the angular frequency; *S*_xy_(*ω*) is the cross-spectra of two discrete time signals; *S*_xx_(*ω*) and *S*_yy_(*ω*) are the spectra of *x*(*t*) and *y*(*t*), respectively; and *K*_xy_(*ω*) and *Q*_xy_(*ω*) are co- and quad-spectra, respectively^19^. Here, *x*(*t*) and *y*(*t*) denote the variation in the VLF/LF amplitudes for each path, and *B*_H_ or *E*_z_, respectively.

*K*_xy_(*ω*) and *Q*_xy_(*ω*) are the real and imaginary parts of *S*_xy_(*ω*), respectively, and are calculated as follows:

2 $$ {\text{S}}_{{{\text{xy}}}} \left( {\upomega } \right) = {\text{K}}_{{{\text{xy}}}} \left( {\upomega } \right) - {\text{iQ}}_{{{\text{xy}}}} \left( {\upomega } \right) $$

## Data Availability

VLF/LF transmitter signal data were downloaded from https://pparc.gp.tohoku.ac.jp/research/vlf/. Magnetic field data at Kakioka are available from https://www.kakioka-jma.go.jp/en/index.html. The SORATENA atmospheric pressure data in Japan are available upon request at https://global.weathernews.com/news/16551/.
